# An up to date on clinical prospects and management of osteoarthritis

**DOI:** 10.1016/j.amsu.2021.103077

**Published:** 2021-11-19

**Authors:** Mudasir Maqbool, Ginenus Fekadu, Xinchan Jiang, Firomsa Bekele, Tadesse Tolossa, Ebisa Turi, Getahun Fetensa, Korinan Fanta

**Affiliations:** a Department of Pharmaceutical Sciences, University of Kashmir, Hazratbal Srinagar, 190006, Jammu and Kashmir, India; b School of Pharmacy, Faculty of Medicine, The Chinese University of Hong Kong, Shatin, N.T, Hong Kong; c School of Pharmacy, Institute of Health Sciences, Wollega University, Nekemte, Ethiopia; d Department of Pharmacy, College of Health Science, Mettu University, Mettu, Ethiopia; e Department of Public Health, Institute of Health Sciences, Wollega University, Nekemte, Ethiopia; f School of Nursing and Midwifery, Institute of Health Sciences, Wollega University, Nekemte, Ethiopia; g School of Pharmacy, Institute of Health Science, Jimma University, Jimma, Ethiopia

**Keywords:** Clinical prospects, Inflammatory cytokines, Pain pathology, Pharmacotherapy, Osteoarthritis

## Abstract

The rising prevalence of osteoarthritis (OA) in the general population has necessitated the development of novel treatment options. It is critical to recognize the joint as a separate entity participating in degenerative processes, as well as the multifaceted nature of OA. OA is incurable because there is currently no medication that can stop or reverse cartilage or bone loss. As this point of view has attracted attention, more research is being directed toward determining how the various joint components are impacted and how they contribute to OA pathogenesis. Over the next few years, several prospective therapies focusing on inflammation, cartilage metabolism, subchondral bone remodelling, cellular senescence, and the peripheral nociceptive pathway are predicted to transform the OA therapy landscape. Stem cell therapies and the use of various biomaterials to target articular cartilage (AC) and osteochondral tissues are now being investigated in considerable detail. Currently, laboratory-made cartilage tissues are on the verge of being used in clinical settings. This review focuses on the update of clinical prospects and management of osteoarthritis, as well as future possibilities for the treatment of OA.

## Highlights

• Osteoarthritis (OA) is a general term that incorporates several different joint diseases.
• The exact pathophysiology of OA remains unclear.
• OA is incurable because there is currently no medication that can stop or reverse cartilage or bone loss.
• Nonsteroidal anti-inflammatory drugs are the most frequently prescribed medications to alleviate arthritic discomfort.
• Stem cell therapies to target articular cartilage and osteochondral tissues are now under investigation.

## 1 Introduction

Osteoarthritis (OA) is a general term that incorporates several different joint diseases. OA's main effects include cartilage degradation, acute and chronic synovial inflammation, subchondral bone alteration, the presence of osteophytes, and changes in synovial fluid (SF) [1]. The first studies on OA were conducted about 130 years ago, and we now recognize OA as a multifactorial, complex disorder. OA was once believed to be a degenerative condition, but it is now known to have an infectious cause, as well as a metabolic etiology, according to recent research [2,3].

A declining younger workforce would sustain an aging population, resulting in a demographic problem around the world. According to predictions, Asia's elderly population will double by approximately 6.8% in 2008 to 16.2% in 2040 [4]. By the age of 65, most people have radiographic proof of OA, and by the age of 75, about 80% of people have radiographic proof of OA. Despite its public health implications, epidemiologists are still affected by OA [5]. For example, OA is predicted to be the fourth major disability issue in India's elderly population, with a prevalence of up to 56.6% [6]. In the United States, osteoarthritis affects more than 32 million people, although statistics vary according to how the disease is interpreted. Osteoarthritis is the most prevalent articular disease worldwide. Estimates of its prevalence vary significantly among populations [7,8]. The present review aimed to provide an update on the pathology and drug regimens of OA and the latest advancements in pharmacotherapy for OA.

## 2 OA's inflammatory pathology

Cytokines are a group of secreted polypeptides that are essential for the initiation of inflammation. Additionally, these cytokines are divided into those released in response to acute or chronic inflammation [[9], [10], [11]]. Several cytokines are important for the mediation of acute inflammatory reactions, including TNF-α, IL-1, IL-11, IL-8, and IL-6. TNF-α and IL-1 (α and β) are two of the most active inflammatory agents found in the body. Chronic inflammation can occur as a result of acute inflammation, which can last for weeks, months, or even years, as the name suggests [[12], [13], [14]].

Cytokines implicated in chronic inflammatory processes are divided into those that contribute to humoral inflammation, such as IL-3, IL-4, IL-5, IL-6, IL-7, IL-9, IL-10, IL-13, and TGF-, and those that contribute to cellular inflammation, such as IL-1, IL-2, IL-3, IL-4, IL-7, IL-9, IL-10, IL-12, and transforming growth factor-beta [15,16]. Another significant cytokine implicated in OA pathology and disease onset is TNF-α [17]. TNF-α stimulates the secretion of proteolytic enzymes by chondrocytes and synovial fibroblasts, which play a key role in apoptotic cell death, inflammation, and matrix degradation. Therefore, the early inflammatory process is initiated by biochemical disturbances and molecular activity, which also determines the extent of the inflammatory response [18,19].

There is evidence that IL-3 is released in response to pathophysiological conditions; however, it is also likely that it regulates bone metabolism in both natural and abnormal circumstances [20]. The pro- and anti-inflammatory effects of IL-6, which is formed by several non-lymphoid and lymphoid cells, as well as chondrocytes and osteoblasts in OA, are well established. IL-6 has the capacity to inhibit catabolic factors implicated in cartilage degeneration while also inducing inflammation, indicating that it has a dual role in disease pathology. The majority of patients with OA have IL-6 found in their SF, which is directly linked to cartilage degradation [21].

Local inflammation and synovial tissue loss are also linked to IL-17, a pro-inflammatory cytokine. The presence of IL-17 in the blood, SF, and synovium biopsy samples in patients with OA is strongly linked to the occurrence of knee OA (increasing KL grade). SF IL-17 may be a valuable biochemical predictor of the incidence and progression of knee OA [[22], [23], [24]].

IL-4 is an anti-inflammatory cytokine that inhibits the growth and effects of IL-1 and TNF-α. IL-4 stimulates neuronal responses by binding to multimeric receptors. It inhibits chondrocyte development of metalloproteinases in response to IL-1 in vitro and has chondroprotective properties [25,26].

IGF-1 is essential for articular cartilage homeostasis because it activates chondrocytes to generate matrix proteins, inhibits their degradation, and prevents cell death. Increases in IGF-1 activity are assumed to be an effort by cartilage to re-establish homeostasis in OA, but they are mostly unsuccessful [[27], [28], [29], [30]].

The exact pathophysiology of OA remains unclear. Oxidative stress and chronic inflammation in the synovium, on the other hand, are known to intensify cartilage degeneration and synovitis, an acute condition of inflammation. SF proteome/cytokine profiling, on the other hand, reveals the existence of both pro- and anti-inflammatory causes. The proportion of inflammatory factors is thought to increase as the disease progresses, resulting in increased cartilage degradation [31,32]. It is interesting to see how these causes affect cartilage loss as well as acute synovial inflammation [32].

## 3 Pathology of pain in OA

There is no direct link between the degenerative phase of cartilage in OA and pain [33,34]. Nerve fibers provide information to the synovial membrane, ligaments, outer menisci, and subchondral bone. Chronic pain has an emotional dimension, although the main mechanisms of pain signal processing are unclear [35,36]. Mental health problems are common comorbidities among patients with OA, but they have been linked to severe pain [37].

Pain is a complex neurological syndrome that affects patients with OA and is believed to be regulated by both nociceptive and neuropathic pathways [38]. As nociceptors are stimulated mechanically or chemically in response to local tissue damage, a pain signal is transmitted from the joint to the dorsal root ganglion of the spinal cord and then up the spinothalamic tract to cortical processing centers [36,39]. Chemical impulses are produced when a tissue is damaged, causing physiological pain and potentially sensitizing nociceptors [35,38].

Nerve dysfunction causes neuropathic pain, which can be detected in people with OA using questionnaires that measure the type and intensity of pain stimuli [40,41]. Neurotransmitters are secreted into the spinal cord as a result of prolonged stimulation of pain fibers in OA and other chronic pain disorders, resulting in increased synaptic regulation and peripheral and central pain perception [35,38]. Cytokines can activate neurons directly or indirectly, slowing their firing rate and causing changes [33,42].

Dormant silent nociceptors in a joint may be activated by inflammatory mediators. Afferent terminals in the joints contain chemical mediators called neuropeptides, which can induce neurogenic inflammation [33,42]. Furthermore, neurogenic inflammation is thought to cause joint damage by encouraging and exacerbating the inflammatory response [43].

## 4 Management of OA

### 4.1 Non-pharmacological management

There is convincing evidence that standard exercise programs can significantly reduce pain and enhance physical function in patients with knee OA. As a result, acupuncture, aquatic exercise, electroacupuncture, inferential current, kinesio taping, manual therapy, moxibustion, pulsed electromagnetic fields, tai chi, ultrasound, yoga, and whole-body vibration all have silver proof backing them up [44]. Surgical management is also alternative non pharmacological treatment among eligible patients. Some non-pharmacologic rehabilitative interventions are summarized as follows.

#### 4.1.1 Exercise

Almost all international recommendations recommend exercise as a first-line treatment for patients with OA. Exercise is recommended as a recovery option for patients with knee OA who want to improve their physical function while also reducing pain [45,46]. Exercise, on the other hand, was discovered to have a pain-relieving effect similar to that of simple analgesia or non-steroidal anti-inflammatory drugs, but with fewer side effects. Although previous research indicated that exercise had limited pain-relieving and physical-functioning benefits, a new study discovered that exercise had a significant positive effect on symptomatic hip OA patients [46,47].

Aerobic training also improves resistance training's muscle-strengthening properties, which, as previously stated, has beneficial effects [48,49]. Patients should be informed about the disease's everyday variability and how over-exercising can affect them, which can manifest as increased pain during operations lasting more than 1 or 2 h, edema, exhaustion, and muscle fatigue [50,51]. However, the patient's education should not instill fear of activity, as this is often linked to a poor care response [49].

#### 4.1.2 Physical therapy (heat and cooling)

Heating decreases discomfort while also increasing the expression of heat shock protein 70 (HSP 70), which has a relaxing and calming effect on OA patients. HSP 70 is involved in cartilage defense, reducing inflammation, and preventing chondrocyte apoptosis [52,53]. To date, pain relief has been the only benefit of shallow cold therapy [54].

#### 4.1.3 Neuromuscular electrical stimulation (NMES)

Neuromuscular electrical stimulation (NMES) is also controversial in women with moderate to severe osteoarthritis of the knee. NMES has been shown to have no effect on quadriceps muscle strengthening [50]. The Cochrane Review found no improvement in isometric resistance despite signs of improved quadriceps muscle activation [50,55].

#### 4.1.4 Pulsed electromagnetic field therapy (PEMF)

According to a meta-analysis of the PEMF RCT, PEMF implementation in OA management greatly increased the patient's daily operation. There was no increase in pain or stiffness, on the other hand [56,57]. The OA guidelines list PEMF therapy as an alternative to other treatment options. Physiotherapy and other less expensive methods of medication have similar benefits to PEMF. Consequently, PEMF can be replaced by physical therapy, at least in some cases [58].

#### 4.1.5 Transcutaneous electrical nerve stimulation

Transcutaneous electrical nerve stimulation (TENS) is a pain-relieving procedure that can be used to treat a wide range of medical problems. It is often used for severe pain or in cases where pharmacotherapy fails [59,60]. TENS has also been shown to be successful in the treatment of OA. TENS associated with exercise reduced pain and increased quadriceps muscle activation, resulting in better physical activity [50].

#### 4.1.6 Low-level laser therapy

Low-level laser therapy (LLL) combined with exercise has been shown to alleviate pain while improving mobility and movement in patients with knee OA. Radiation also increases local microcirculation and is highly recommended as a supplement to other treatments for OA [50,61].

#### 4.1.7 Massage

Massage for 60 min a week after eight weeks of therapy increased pain management and the Western Ontario and McMaster Universities Arthritis Index (WOMAC) functionality scores [62,63]. There was no discernible difference between the experimental and normal treatments after 24 weeks. The repositioning of the affected knee joint was not facilitated by relaxing massage of the quadriceps, gracilis, femoris, sartorious, and hamstrings [50,64].

#### 4.1.8 Acupuncture

Despite its efficacy, the use of acupuncture in the treatment of OA requires further research. Any of the effects may be due to the patient's anticipation, the placebo effect, or even pain relief, both of which contribute to improved posture. It also depends in part on the acupuncturist, who may or may not have prior experience. As a result, more research should be conducted to determine its true efficacy using a double-blind design, so that its use can be checked [50,65].

#### 4.1.9 Assistive devices

##### 4.1.9.1 Canes

Patients with OA frequently use canes to aid in mobility. When patients first use it, their energy expenditure tends to increase. However, after one month, the benefits of using canes could be seen in the form of significant pain reductions and a return to normal energy expenditure because of the adaptation. The cane should be used contra-laterally to significantly reduce the burden on the affected knee while maximizing the benefit [50,66].

##### 4.1.9.2 Braces and insoles

Lateral wedges reduce the knee-ground response force lever arm in patients with medial knee OA, which tends to be the primary cause of load-reduction effects. The impact of valgus knee braces and lateral wedged insoles on biochemistry and health outcomes were contrasted in a recent study [67,68]. Both therapies can help delay the progression of the disease by reducing knee loading in patients with OA. Action levels are raised when valgus unloader braces are worn [66,69].

### 4.2 Pharmacological management

**Fig. 1:**
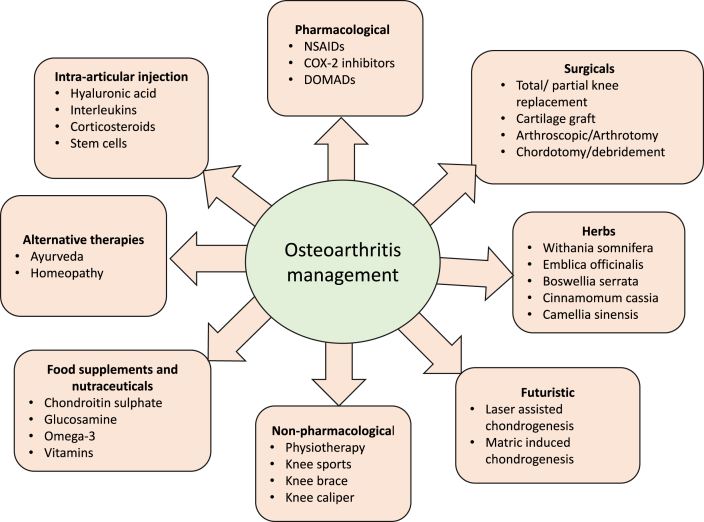
Holistic therapies for the management of osteoarthritis.

The primary goal of pharmacological OA therapy is to alleviate the symptoms of OA. Nonsteroidal anti-inflammatory drugs (NSAIDs) and analgesics are the most frequently prescribed medications in this category. Topical and intra-articular therapies have also been used [70,71]. Palliative pain management is the primary focus of current OA management strategies. Joint replacement surgery has been shown to relieve the disease's excruciating and disabling symptoms in acute circumstances. Currently, no treatments are available to slow or reverse the development of OA [72,73]. Therapeutic solutions for OA are divided into multiple groups, as shown in Fig. 1. The existing care strategies for OA are directed mainly at reducing pain and discomfort, maintaining joint stability, and avoiding function loss [73,74].

Patient screening, diet, yoga, weight loss, acupuncture, and physical rehabilitation, which include thermotherapy, transcutaneous electrical nerve stimulation (TENS), and short-wave diathermy, are all non-pharmacological treatments [75,76].

#### 4.2.1 NSAIDs and analgesics

NSAIDs may be used to help with other conditions, including joint pain. Oral analgesics, including acetaminophen, ibuprofen, diclofenac, and cyclooxygenase type 2 (COX-2) antagonists, as well as intra-articular corticosteroids, are often used to alleviate arthritis discomfort [77,78].

#### 4.2.2 Topical agents

Among them are topical lidocaine, NSAIDs, and capsaicin. By maximizing local absorption, thus minimizing systemic toxicity, topical application of NSAIDs decreases the harmful effects of oral medications [79,80].

#### 4.2.3 Intra-articular therapy

OA is also treated with corticosteroid injections into articular cartilage. Methyl prednisolone acetate, betamethasone acetate/betamethasone sodium phosphate, triamcinolone acetonide, triamcinolone hexacetonide, and betamethasone dipropionate/betamethasone sodium phosphate are among the most frequently prescribed preparations [81,82].

#### 4.2.4 Hyaluronic acid

Hyaluronic acid is a structural polysaccharide present in the cartilage extracellular matrix that plays a crucial role in the development of SF [83]. It is important to maintain the viscoelastic properties of SF because it functions as a lubricant and shock absorber [84].

#### 4.2.5 Anti-cytokine therapy

Chondrocytes and synoviocytes, such as immune cells, can produce a variety of pro-inflammatory cytokines. Many cytokines are involved, but tumor necrosis factor-alpha (TNF-α) is required for the initiation and regulation of inflammatory cascades. TNF-α promotes the development of cytokines and tissue-degrading enzymes such as matrix metalloproteinases (MMPs) [85]. TNF-α is a cytokine produced by monocytes, macrophages, T and B cells, as well as synoviocytes, and its levels increase as the condition worsens. TNF-α antibodies have been shown to significantly minimize joint inflammation and pain [86,87].

Infliximab, golimumab, etanercept, certolizumab pegol, and adalimumab are five anti-TNF agents that have been documented for the treatment of rheumatoid arthritis. The complementarity-determining region of a murine anti-human IL-6R (IL-6 receptor) antibody was combined with human IgG1 to create this humanized monoclonal antibody. Tocilizumab is an anti-inflammatory medication that prevents the activation of the IL-6 receptor [88,89]. Anakinra blocks the biological function of naturally occurring IL-1by competitively inhibiting IL-1 binding to IL-1R, which is expressed in a variety of tissues and organs [90].

#### 4.2.6 Omega-3 fatty acids as dietary supplements

Eicosanoids are mediators as well as regulators of inflammation, and PUFAs are essential fatty acids that produce them. Omega-3 PUFA therapy reduced pro-inflammatory factors and improved anti-inflammatory markers in preclinical and clinical trials [91,92]. In cartilage cell cultures, omega-3 PUFAs have also been shown to inhibit the transcription of essential matrix degradation enzymes and cytokines [93].

Curtis et al. examined how omega-3 and omega-6 fatty acids affect the history of human OA cartilage explants. These findings indicate that omega-3 supplementation can fully suppress the expression of ADAMTS-4, MMP-13, and MMP-3 in the body, as well as other pro-inflammatory mediators (5-lipoxygenase, cyclooxygenase 2, 5-lipoxygenase–activating enzyme, tumor necrosis factor, and interleukin-1) [94].

Another study established that supplementation with EPA and DHA blocked the deterioration of cartilage caused by IL-1β [95]. The involvement of EPA and DHA was found to inhibit the release of sGAGs from cartilage explants induced by IL-1β. Additionally, fish oil supplementation in combination with NSAIDs has been shown to alleviate discomfort and enhance the weight-bearing capability of knee cartilage in laboratory dogs [96].

Fritsch et al. (2010) discovered that feeding a fish oil-rich diet to dogs with OA for 12 weeks reduced caporofen (NSAID) dosage significantly compared to a control diet. Omega-3 fatty acid supplementation was used to treat OA-prone and OA-resistant guinea pig strains using a random OA model in a significant trial [97]. When OA-prone and-resistant strains were compared, OA markers such as lysyl-pyridinoline, active MMP-2, and complete collagen cross-links were altered. When compared to a non-pathological strain fed an omega-3 diet, subchondral bone parameters such as calcium to phosphate ratios and epiphyseal bone density improved significantly [97,98].

Omega 3 fatty acids have been shown to have anti-inflammatory properties in a variety of animal models and clinical trials [99]. Taking an omega-3 fatty acid supplement has been shown in some trials to significantly improve OA symptoms in the knee cartilage and the lower portion of the femur (one of the vertebrae) [100,101].

#### 4.2.7 Herbs and Ayurvedic formulations

Although several herbs have been used in Ayurveda for many years, many of them are commonly used in human remedies; their use in human beings is assumed to be completely safe [102,103]. However, one laboratory trial of these analgesic combinations was shown to have the ability to alleviate OA pain, while others were shown to slow cartilage deterioration, in which excessive Vata energy causes pain, immobility, and rigidity of the limbs [104,105].

Over the last several years, there has been an increased focus on the molecular mechanisms of how herbal drugs work in the body. The ancient Ayurvedic formulations Triphala churna and Triphaghula, along with Balaraja and Dashmoolasa, are used to treat the disease [105,106]. According to ancient texts, triphala and its components have anti-inflammatory, antioxidant, cytoprotective, and rejuvenating effects (Rasayana) [107,108].

In pharmacological research, triphala extract has been shown to have free radical scavenging properties and to minimize the damage caused by oxidative stress. Triphala has also been shown to scavenge nitric oxide (NO) in vitro, as well as exert anti-inflammatory and anti-arthritic effects [109,110]. Triphala reduces inflammatory mediator levels and inhibits lipid peroxidation. Dashamoola formulations are made up of the roots of ten plants that are also useful for vata-roga, as the name suggests. Dashamoolarishta has been used for its anti-inflammatory effects as well as for the relief of inflammation and discomfort associated with arthritis since ancient times [111,112].

In conclusion, Ayurveda mentioned a variety of polyherbal formulations with a long history of medical use in OA, and many of these formulations are successful in reducing tissue inflammation in OA models. As a result, Ayurvedic therapies are emerging as a ray of hope for treating a variety of chronic diseases without the use of potentially risky medications. The current OA drug treatments with the available phenotypes are described in Table 1.

**Table 1:** Current drug treatments for OA with available phenotypes.

Target	Drug categories	Findings	References
A) Treatments for cartilage problems			
Inhibition of cartilage matrix degradation	MMP-inhibitor PG-116800	Termination due to musculoskeletal toxicity	[113, 114]
Cartilage matrix regeneration	Sprifermin (truncated human FGF18)	The thickness of the cartilage of the femorotibial joint has improved.	[113,115]
	BMP-7 or OP-1	Both the BMP-7 and placebo participants experienced pain relief.	[113,116]
**B) Subchondral bone treatment options**			
Bisphosphonates/bone turnover	Zoledronic acid	BML size has shrunk, as has the pain score on the visual analogue scale.	[113,117]
	Risedronate	BMLs reduced discomfort in a patient subgroup.	[113,118]
	AXS-02 (disodium zoledronate tetrahydrate)	BMLs reduced discomfort in a patient subgroup.	[113,119]
Inhibition of bone degradation	Cathepsin K inhibitor MIV-711	Slowdown of bone and cartilage degeneration	[113,120]
**C) Inflammatory-process-targeting therapies**			
IL-1	Anakinra (IL-1 receptor antagonist)	No improvements of OA symptoms	[113,121]
	AMG 108 (fully human monoclonal antibody to IL-1R1)	Minimal clinical benefit	[113,122]
	Lutikizumab (anti IL-1 α/β antibody)	No improvement in synovitis, minimal effect on WOMAC pain score	[113,123]
Tumor necrosis factor-alpha	Adalimumab	There was no difference in discomfort, synovitis, or BMLs	[113,124]
		Increased physical activity and effective pain relief	[113,125]
	Etanercept	There is little pain relief, and MMP-3 concentration in the body was decreasing.	[113,126]
	Infliximab	Treatment of recent-onset RA patients slowed the development of hand OA.	[113,127]
Toll-like receptor 7/9	Hydroxychloroquine	Efficacy was not shown.	[113,128]
I-kB kinase	SAR113945 (I-kB kinase inhibitor)	No superior efficacy	[113,129]
p38 MAP kinase	FX-005	Pain relief superior to placebo	[113]
**D) Treatments that target the causes of pain**			
NGF	Tanezumab (anti-NGF antibody)	Although there has been a small improvement in functional and pain ratings, the rising demand for joint replacement has raised safety concerns.	[113,130]
NGF receptor tropomyosin-related kinase A (TrkA)	Pan Trk inhibitor GZ389988	Short-term moderate pain reduction compared to control	[113,131]
Transient receptor potential vanilloid 1 (TRPV1) receptor	*Trans*-capsaicin (CNTX-4975)	Over the course of 24 weeks, intra-articular CNTX-4975 minimised moderate-to-severe pain relative to placebo.	[113,132]
	Mavatrep (JNJ-39439335)	Significant pain relief and improved function were achieved, but dosage changes were needed due to altered heat sensitivity and the subsequent thermal burns.	[113,133]
Kappa-opioid receptor	Selective agonist CR845	Dose-dependent pain relief is more successful in patients with hip OA.	[113,133]
Alpha calcitonin gene-related peptide	Galcanezumab (LY2951742)	The study was halted due to insufficient efficacy.	[113,134]
Imidazoline receptor I2	CR4056 (receptor ligand)	Analgesia that works, particularly in men and patients with metabolic syndrome who are overweight.	[113,135]
**E) Medications for the metabolic syndrome**			
Cox-2 and T2DM	Cox-2 inhibitor and metformin	Lower rate of receiving joint replacement surgery	[113,136]
HMG-CoA-Reductase	Statins: simvastatin, atorvastatin, atorvastatin calcium, lovastatin, fluvastatin sodium, nystatin, pravastatin, pravastatin sodium, rosuvastatin, and rosuvastatin calcium	Regardless of other possible confounding variables, radiological deterioration over 3 years	[113,137]
	Statins: atorvastatin, fluvastatin, pravastatin, rosuvastatin, or simvastatin	No protective effect of statins on the risk of developing hand OA	[113,137]
	Statins: pravastatin, simvastatin, fluvastatin, rosuvastatin, lovastatin, and atorvastatin	The use of statins is not linked to a lower risk of hip or knee OA consultations or surgeries.	[113,138]

### 4.3 Surgical management

Many people with osteoarthritis develop to severe joint degeneration in the absence of disease-modifying medication. As a result, surgery is crucial in the treatment of OA. Biomaterials and tissue engineering advancements will continue to open up interesting new possibilities for integrating surgical techniques into OA treatment. Weak study designs and small samples limit the data supporting the utility of various surgical procedures. Scientific breakthroughs in the field will require extensive examinations of the efficacy and cost effectiveness of surgical approaches to OA therapy [139,140].

Surgery could be used if alternative treatments have failed to alleviate the patient's symptoms. Consistent pain and disability despite conservative treatment are a well-accepted indication for surgery. Total joint replacement is the most effective surgical procedure, with good patient outcomes after hip, knee, and shoulder replacements. There are a variety of prosthetic devices available, but there are no controlled trials comparing them. Most contemporary joint prosthesis are expected to last 15–20 years for most patients [141,142]. Other surgical treatments for osteoarthritis exist, but none have shown to be as effective as total joint replacement. Arthroscopic debridement for osteoarthritis of the knee has continuously failed to show a benefit over maximal medical therapy combined with physical therapy in randomized trials [143].

For severe clinical illness that has not responded to conservative treatment, joint replacement surgery should be considered. In persons who are getting physical and medicinal therapy for knee osteoarthritis, arthroscopic operations have not offered any further benefit [[143], [144], [145]]. Surgery should be considered if conservative therapy fails. Arthroscopy, cartilage repair, osteotomy, and knee arthroplasty are surgical treatments for knee OA. The location, stage of OA, comorbidities, and patients suffering on the other side all play a role in determining which of these operations is most suited. Arthroscopic lavage and debridement are frequently performed; however they have no effect on disease development. Unicompartmental (partial) knee arthroplasty or unloading osteotomy may be explored if OA is restricted to one compartment. Because of the dangers and limited durability of complete knee replacement, they are indicated in young and active patients. In older patients with advanced knee OA, total knee arthroplasty is a routine and safe procedure [146,147].

## 5 Available gaps and future therapies

In the pathogenesis of osteoarthritis, the matrix-degrading enzymes MMP-13 and ADAMTS-5 play a role. In a mouse model of osteoarthritis, CL82198, an inhibitor of MMP-13, inhibited chondrocyte apoptosis and delayed cartilage degradation [148]. However, such findings in humans are yet to be verified. Due to musculoskeletal toxicity, the only MMP blocker clinical trial (PG-116800) has been halted [149]. MMP-1 and MMP-7 are two MMPs that are thought to play a role in the progression of musculoskeletal toxicity, and PG-116800 binds to both MMPs [150].

Further research is needed to fully evaluate the efficacy and safety of MMP inhibitors. Chen et al. examined the use of an ADAMTS-5 blocker to treat osteoarthritis in mouse knee joints [151]. After eight weeks, a combination of an ADAMTS-5 inhibitor (114810) and hyaluronic acid hydrogel reduced cartilage degeneration and allowed cartilage regeneration, implying that ADAMTS-5 may be a target for osteoarthritis treatment.

ADAMTS-5 activation has also been linked to Syndecan-4 [152]. As a result, using a syndecan-4-specific antibody inhibits the activation of ADAMTS-5, thereby slowing the progression of osteoarthritis. Although articular cartilage has been the focus of most clinical target trials, subchondral bone can also be implicated in the disease phase. TGF-β has been established as a key player in subchondral bone formation.

Zhen et al. discovered TGF-β activation in the subchondral bone in response to changed mechanical loading in a mouse osteoarthritis model with anterior cruciate ligament transection [153]. Inhibition of TGF-β activity in the subchondral bone also decreased articular cartilage degeneration. Furthermore, the Wnt/β-catenin signalling pathway may be a viable option [154]. Dkk-1 suppression was found to improve osteoarthritis in a mouse model in a recent report [155]. These results emphasize the importance of treating osteoarthritis as a whole-joint condition.

Recent developments in OA pathology have shown the critical functions of several novel pathways that can be addressed. However, because OA is a diverse illness, a single medication targeting a specific joint tissue may be ineffective, and no “one-size-fits-all” drug or therapy will ever be discovered. Timely changes in disease development, such as the switch from high bone turnover in early OA to lower bone turnover later, or changes in pain type, necessitate accurate knowledge of the underlying molecular alterations.

In the future, selecting appropriate medication for specific disease time-points could aid in tailoring personalized treatment regimens for each patient. Furthermore, OA may present with overlapping endotypes, such as an inflammatory pain endotype that could benefit from a combination of pain and inflammation-fighting medications [113]. Over the next few years, several prospective therapies focusing on inflammation, cartilage metabolism, cellular senescence, subchondral bone remodelling, and the peripheral nociceptive pathway are predicted to transform the OA therapy landscape.

The most promising treatments are those that target articular cartilage molecular processes [[156], [157], [158]]. The development of new therapeutic options for OA will require better knowledge of the disease. However, according to the literature, traditional repair strategies continue to predominate in clinics. The first treatment for OA patients is mainly conservative management and pharmaceutics. These methods can help relieve pain and improve joint functionality and may even help to slow the progression of OA.

There are various options for chondrocytes and stem cells, such as mesenchymal stem cells and adipose-derived stem cells, as cell sources. Concerns regarding efficient supply, safety, the creation of non-specialized tissue, cancer, and legal difficulties must be addressed [159,160]. Moreover, the standardization of the method, which includes biomaterial synthesis, cell separation, and maintenance, remains unresolved. Gene therapy could be another advanced treatment option for OA.

Furthermore, reversing the derailment of cellular mechanoreceptive pathways may open new avenues for preventing structural tissue degradation. Although many issues remain, these approaches show enormous potential for the future; nonetheless, success will only be achieved if efforts are united. To establish a valid strategy for OA in healthcare, researchers and doctors must collaborate.

## 6 Conclusion

Osteoarthritis (OA) severely restricts the everyday activities of senior citizens. The frequency and prevalence of OA are anticipated to double over the next decade. Patients with osteoarthritis seek medical help because of their suffering. While IL-6 can suppress catabolic pathologies associated with cartilage degeneration, it can also drive cartilage degeneration. OA has seen quite a bit of change over the past few years. Nonnarcotic analgesics, such as nutraceuticals and intraarticular drugs, are some of the new and more efficacious agents, such as those in the market.

The more advanced the understanding and use of nonpharmacological interventions (such as patients, exercise, and weight reduction when needed), and the more frequently they are applied, the better the results. In most patients, pain relief and the ability to move or use their joints can be obtained using an integrated approach. When it comes to the many routes that are used, a single-targeted approach is not likely to address the problem. Thus, as with other long-term diseases, OA treatment has the potential to improve.

## Ethical approval

Not applicable.

## Source of funding

This research did not receive any specific grant from funding agencies in the public, commercial, or not-for-profit sectors.

## Authors’ contribution

MM, GF* and XJ drafted the review and contributed to the writing of the manuscript. FB, ET, TT, GF and KF provided critical input and revised the manuscript. All authors critically revised the manuscript, accountable for all aspects of the work and approved the final version to be published.

## Consent for publication

Not applicable. No individual personal details, images, or videos were being used in this study.

## Registration of research studies

1. Name of the registry: Not applicable
2. Unique Identifying number or registration ID: Not applicable
3. Hyperlink to your specific registration (must be publicly accessible and will be checked): Not applicable

## Provenance and peer review

Not commissioned, externally peer reviewed.

## Guarantor

Ginenus Fekadu.

## Availability of data and materials

The datasets used and/or analyzed during the current study were included in the published article.

## Declaration of competing interest

The authors declare that they have no competing interests.

## Abbreviations

AC: Articular Cartilage
IL: Interleukin
LLL: Low-level laser therapy
MMPs: Matrix metalloproteinase
NMES: Neuromuscular electrical stimulation
NSAIDs: Nonsteroidal anti-inflammatory drugs
OA: Osteoarthritis
SF: Synovial fluid
TENS: Transcutaneous electrical nerve stimulation
TNF-α: Tumour necrosis factor-alpha
WOMAC: Western Ontario and McMaster Universities Arthritis Index
